# Interplay between triglyceride-glucose index and cardiovascular events in hypertensive patients with controlled blood pressure (SBP<140 mmHg): insights from the SPRINT trial

**DOI:** 10.3389/fendo.2025.1508667

**Published:** 2025-07-16

**Authors:** Xiaoling Chen, Lili Wang, Yingying Jiang, Wenyi Tang, Jun Xiao, Wuquan Deng, Xiangpeng Ren, Chuanwei Li

**Affiliations:** ^1^ Department of Cardiology, Chongqing Emergency Medical Center, (Center Hospital of Chongqing University), Chongqing University, Chongqing, China; ^2^ Department of Cardiology, Beijing Anzhen Hospital Affiliated to Capital Medical University, Beijing, China; ^3^ Department of Clinical Data Research, Chongqing Key Laboratory of Emergency Medicine, Chongqing Emergency Medical Center, (Center Hospital of Chongqing University), Chongqing University, Chongqing, China; ^4^ Department of Endocrinology, Chongqing Emergency Medical Center (Center Hospital of Chongqing University), Chongqing University, Chongqing, China; ^5^ Department of Biochemistry, Medical College, Jiaxing University, Jiaxing, China

**Keywords:** hypertension, insulin resistance, TyG index, cardiovascular disease, blood pressure control

## Abstract

**Introduction:**

Hypertension significantly contributes to cardiovascular events and global all-cause mortality. The triglyceride-glucose (TyG) index, amarker of insulin resistance (IR), is an established risk factor for cardiovascular disease (CVD) events. This study examined the relationship between the TyG index and cardiovascular events in patients with controlled hypertension (SBP <140 mmHg).

**Methods:**

We performed a post-hoc analysis of data from the Systolic Blood Pressure Intervention Trial (SPRINT), involving 9,323 participants with controlled hypertension. The triglyceride-glucose (TyG) index served as a surrogate marker for IR. Cox restricted cubic regression analysis and multivariate Cox regression models were employed to investigate the association between the TyG index and CVD outcomes, adjusting for established cardiovascular risk factors. The impact of intensive versus standard BP control on CVD risk associated with high IR levels were also analyzed.

**Results:**

Over a median follow-up of 3.33 years, 725 CVD events were recorded. The TyG index was independently linked to a heightened risk of CVD events,with the highest quartile (Q4:8.93≤TyG<12.47) exhibiting a significantly greater risk (HR = 1.57, 95% CI: 1.18–2.08) compared to the lowest quartile (Q1: 6.74 ≤ TyG < 8.21). The significantly trend was seen only in the standard treatment group (p for trend = 0.001).

**Discussion:**

The TyG index is a robust predictor of CVD events in patients with controlled hypertension, and a stronger association between the TyG index and CVD risk was seen in the standard treatment group, but not in the intensive 1treatment group.

**Clinical trial registration:**

http://www.clinicaltrials.gov, identifier NCT01206062.

## Introduction

Hypertension is the primary cause of cardiovascular events and mortality globally, presenting a significant and growing challenge for global public health (1), and it affects approximately 31.1% of the global adult population (2). Despite the widespread administrate of antihypertensive drugs, the morbidity of hypertension continues to rise. A 5 mm Hg reduction in systolic blood pressure (SBP) typically lowers the risk of major cardiovascular events by approximately 10%, however, the ideal extent of blood pressure (BP) reduction and target BP levels should be tailored according to individual cardiovascular risk stratification (3).

Hypertension, obesity, and dyslipidemia commonly coexist as components of metabolic syndrome and are linked to increased insulin resistance (IR) (4). However, managing hypertension can affect hyperinsulinemia and the progression to severe IR status, including diabetes mellitus (DM) (5). The triglyceride-glucose (TyG) index, described as a simple, convenient, and cost-effective method, has been proposed as a surrogate marker for IR, demonstrating a strong correlation with both the insulin clamp technique and the homeostatic model assessment of IR (HOMA-IR) (6, 7). Therefore, we utilized the TyG index as a practical and validated surrogate for IR, chosen for its accessibility and strong correlation with HOMA-IR, and the euglycemic clamp in large epidemiological studies like SPRINT, where direct insulin measurements were unavailable.

Actually, previous studies have demonstrated the associations between the TyG index and the incidence of cardiovascular disease(CVD) (8), as well as cardiovascular and all-cause mortality (9, 10). In addition, BP status partially mediated the relationship between IR and both cerebral small vessel disease (11) and CVD (12). In recent years, intensive BP control has emerged as a new trend in hypertension treatment, particularly for high-risk populations. While the ACCORD study (13) indicated that intensive BP control (SBP<120mmHg) did not lower the risk of CVD events in patients with hypertension and diabetes, later studies like the SPRINT trial (14) (excluding DM patients), and STEP studies (15) (including approximately 20% of DM patients, intensive BP): confirmed that intensive BP control (SBP<120mmHg in SPRINT trial, SBP<130mmHg in STEP studies) could indeed reduce the risk of CVD events. These findings suggest that variations in glucose metabolism status may result in different responses to intensive BP control.

In the SPRINT study, high CVD risk participants receiving intensive treatment experienced lower rates of major adverse cardiovascular events and reduced all-cause mortality (14), with a slightly higher risk of new-onset DM compared to those receiving standard treatment (SBP<140 mmHg) (16). We hypothesized that intensive BP lowering might ameliorate endothelial dysfunction or inflammation pathways exacerbated by insulin resistance, thereby mitigating CVD risk. It remains unclear whether intensive controlled BP status can reduce the risk of CVD endpoints associated with IR in hypertension. Therefore, this study aimed to assess the correlation between the TyG index and CVD event incidence in patients with controlled hypertension (SBP <140mmHg) through a *post-hoc* analysis of the SPRINT study, and to examine whether two different BP control goals affect clinical outcomes at varying IR levels.

## Methods

### Data reproducibility statement

All anonymized data and materials from the SPRINT study are publicly available through BioLINCC and can be accessed at https://biolincc.nhlbi.nih.gov/studies/sprint/?q=SPRINT.

### Study design and participants

**Figure 1:**
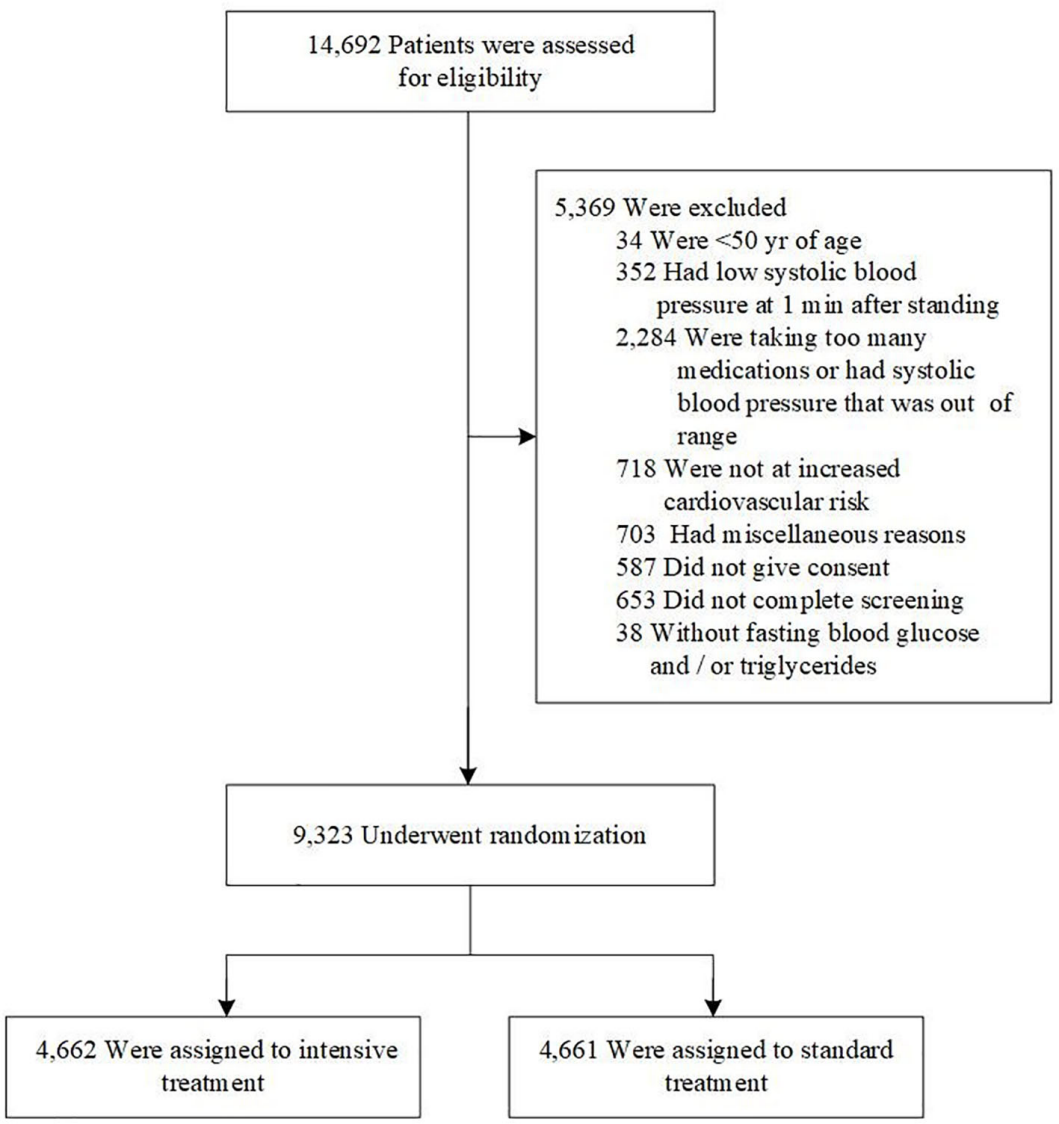
Eligibility, randomization, and follow-up.

SPRINT, a randomized controlled open-label trial, enrolled participants and concluded in August 2015. The design, methods, and data collection details of the SPRINT study cohort have been previously published (14, 17). In brief, the SPRINT study recruited 9,361 patients aged 50 years and older between November 2010 and March 2013 in the United States. The randomized, controlled, open-label trial was conducted at baseline and follow-up. All participants gave written informed consent.Participants with missing fasting blood glucose (FBG) or triglycerides (n=38) data were excluded from this analysis. Thus, 9,323 eligible participants were finally included in current study (**Figure 1**).

### Data collection and definitions

Sociodemographic information, smoking status, Framingham risk scale score, personal medical history, CVD, chronic kidney disease (CKD) history, and clinical and laboratory data were collected. The latter included total cholesterol (TC), low-density lipoprotein cholesterol (LDL-C), triglycerides, high-density lipoprotein cholesterol (HDL-C), FBG, blood urea nitrogen (BUN), estimated glomerular filtration rate (eGFR), and serum creatinine (SCr), which were collected at baseline. Body mass index (BMI) was determined by dividing weight (kg) by height squared (m²). BP was measured using the average of three readings taken at 1-minute intervals with an automated oscillometric device (Model HEM-907XL; Omron Healthcare) after the participant had been seated for 5 minutes. The TyG index was calculated using the formula: ln [fasting triglycerides (mg/dl) * fasting glucose (mg/dl)/2] (18).

### Study outcomes

The outcomes of interest were CVD events, including nonfatal myocardial infarction, acute coronary syndrome without myocardial infarction, nonfatal stroke, nonfatal acute decompensated heart failure, and cardiovascular mortality.

### Statistical analysis

All continuous variables were assessed for normality via the Kolmogorov-Smirnov test. Continuous variables with normal distribution were presented as means and standard deviations (SDs), while those with non-normal distribution were reported as medians and interquartile ranges. Categorical variables were expressed as counts (n) and percentages (%). The Kruskal–Wallis H-test or chi-square tests were employed to evaluate group differences based on baseline TyG index quartiles. (Q1, 6.74≤TyG<8.21; Q2, 8.21≤TyG<8.55; Q3, 8.55≤TyG<8.93; Q4 8.93≤TyG<12.47). The Kaplan–Meier curve was used to analyze the cumulative incidence of endpoint events across TyG index groups.

The variance inflation factor (VIF) was employed to assess multicollinearity among covariates. Variables with a VIF≥5, indicating collinearity, were excluded from the model. Cox restricted cubic regression (RCS) analysis was used to investigate the association between the TyG index and outcomes. Cox regression analysis was used to estimate unadjusted and adjusted hazard ratios (HRs) and 95% confidence intervals (CI) for outcomes based on baseline TyG index. The TyG index was included in the model both as a continuous variable and as quartiles. Confounding factors were identified based on statistically significant clinical variables from univariate Cox regression analyses, collinearity diagnosis, and known outcome correlations. SCr (VIF 7.6), TG (VIF 11.94), and TC (VIF 21362) were not included in Model 3 after the collinearity diagnosis. Finally, Model 1 was unadjusted; Model 2 was adjusted for age, gender, and Black race; and model 3 included all variables from Model 2 plus BMI, CVD history, CKD history, Framingham risk scale score, number of antihypertensive drugs, use of aspirin, β-blockers, calcium channel blockers (CCBs), diuretics, stains, angiotensin-converting enzyme inhibitors/angiotensin II receptor blockers (ACEIs/ARBs), smoking status, SBP, diastolic blood pressure (DBP), eGFR, BUN, fasting HDL-C, fasting LDL-C, and FBG. The reference group in all three models was the lowest quartile of the TyG index (Q1). Statistical analyses were conducted using SPSS version 23.0 (SPSS, Chicago, IL) and R version 4.4.0 (R Foundation, Vienna, Austria), with significance set at two-sided p-values < 0.05.

## Results

### Baseline characteristics

**Table 1** displays the baseline characteristics of the analytical cohort, organized by TyG index quartiles. The study analyzed 9,323 patients, 35.5% of whom were female, with a median age of 67.9 years (range 58–68 years). Of the cohort, 2,913 (31.2%) were Black, 2,644 (28.4%) had a history of CKD, and 1,869 (20%) had a history of CVD. All patients were free of documented DM but were at high risk for cardiovascular events, as indicated by a median Framingham risk score of 17. Among the participants, 4,662 individuals (50%) were in the intensive treatment group, and 4,151 individuals (44.5%) had never smoked. The median number of antihypertensive medications used was two, with ACEIs/ARBs being the most common (57.4%), followed by diuretics (43.4%), β-blockers (35.8%), CCBs (9.4%), and α-blockers (7.5%). Significant variations in the distribution of specific drug classes were observed when stratified by the TyG index. Patients in the lower quartile had a higher usage of CCBs and α-blockers (p < 0.001), while those in the higher quartile showed increased use of β-blockers (p < 0.001) and diuretics (p = 0.007).

**Table 1:** Baseline characteristics of total and groups by TyG index quartile.

Variables	Overall (n=9,323)	TyG index quartile				*p* value
		Q1:6.74≤TyG<8.21 (n=2,336)	Q2:8.21≤TyG<8.55 (n=2,326)	Q3:8.55≤TyG<8.93 (n=2,331)	Q4:8.93≤TyG<12.47 (n=2,330)	
Age (years)	63 (58~68)	64 (59~69)	63 (58~68)	63 (59~68)	63 (58~67)	<0.001
Female sex -no. (%)	3,307 (35.5)	889 (26.9)	874 (26.4)	807 (24.4)	737 (22.3)	<0.001
Race no. (%)						
Black	2,913 (31.2)	999 (34.3)	795 (27.3)	619 (21.2)	500 (17.2)	<0.001
Other	6,410 (68.8)	1,337 (20.9)	1,531 (23.9)	1,712 (26.7)	1,830 (28.5)	
CKD history-no. (%)	2,644 (28.4)	593 (22.4)	664 (25.1)	689 (26.1)	698 (26.4)	0.002
CVD history-no. (%)	1,869 (20.0)	464 (24.8)	465 (24.9)	477 (25.5)	463 (24.8)	0.95
Framingham Risk Scale	17 (16-19)	17 (16-18.25)	17 (16-19)	17 (16-19)	18 (16-20)	<0.001
Intensive treatment- no. (%)	4,662 (50.0)	1,187 (25.5)	1,148 (24.6)	1,190 (25.5)	1,137 (24.4)	0.337
BMI	29.00 (25.86~32.89)	28.36 (25.04~32.34)	28.59 (25.64~32.70)	29.16 (26.29~33.07)	29.89 (26.73~33.61)	<0.001
Smoking status-no. (%)						
Never	4,151 (44.5)	1,056 (25.4)	1,064 (25.6)	1,029 (24.8)	1,002 (24.1)	0.257
Former	3,934 (42.2)	968 (24.6)	979 (24.9)	997 (25.3)	990 (25.2)	
Current	1,238 (13.3)	312 (25.3)	283 (22.9)	305 (24.6)	338 (27.3)	
SBP	138 (129~148)	138.5 (130~150)	137 (128~149)	137 (129~147)	138 (129~147)	0.04
DBP	81 (73~88)	81 (73~88)	81 (73~88)	80 (73~88)	82 (74~89)	<0.001
Antihypertensive agents - no./patient (%)						
Number of drugs	2 (1~3)	2 (1~2)	2 (1~2)	2 (1~3)	2 (1~3)	0.17
ACEI/ARB	5,347 (57.4)	1,309 (14.0)	1,331 (14.3)	1,352 (14.5)	1,355 (14.5)	0.443
β-blocker	3,341 (35.8)	742 (8.0)	780 (8.4)	882 (9.5)	937 (10.1)	<0.001
α-blocker	700 (7.5)	211 (2.3)	174 (1.9)	186 (2.0)	129 (1.4)	<0.001
CCB	3,261 (35.0)	879 (9.4)	850 (9.1)	773 (8.3)	759 (8.1)	<0.001
Diuretic	4,043 (43.4)	961 (10.3)	982 (10.5)	1,036 (11.1)	1,064 (11.4)	0.007
Other drugs- no./patient (%)						
Stain	2,124 (22.8)	453 (4.9)	493 (5.3)	566 (6.1)	612 (6.6)	<0.001
Aspirin	4,747 (51.0)	1,172 (12.6)	1,211 (13.0)	1,208 (13.0)	1,156 (12.4)	0.246
eGFR, ml/min/1.73 m^2^	74.78 (62.04~87.65)	76.12 (63.41~89.44)	74.51 (62.8~88.56)	74.91 (61.45~87.04)	73.8 (60.85~86.12)	<0.001
SCr, mg/dl	0.99 (0.85~1.18)	0.99 (0.85~1.17)	0.99 (0.84~1.18)	0.99 (0.85~1.17)	1 (0.85~1.19)	.073
BUN, mg/dl	17 (14~21)	17 (14~21)	17 (14~20)	17 (14~20)	17 (14~21)	.465
Fasting TC, mg/dl	190 (164~217)	182 (157~208)	186 (160~212)	191 (162~216)	202 (176~231)	<0.001
Fasting TG, mg/dl	110 (80~157)	64 (55~72)	91 (83~100)	125 (113~138)	194 (167~239)	<0.001
Fasting HDL-C, mg/dl	49 (42~59)	59 (50~70)	51 (44~60)	48 (42~55)	43 (37~50)	<0.001
Fasting LDL-C, mg/dl	113 (91~137)	108 (87~130)	114 (91~137)	116 (92~138)	116 (93~142)	<0.001
FBG, mg/dl	97 (91~105)	92 (86~98)	96 (90~102)	99 (92~107)	103 (96~113)	<0.001
TyG index	8.59 (8.24~8.97)	7.99 (7.81~8.11)	8.39 (8.3~8.47)	8.72 (8.63~8.82)	9.21 (9.05~9.45)	<0.001

TyG index, triglyceride–glucose index; CVD, cardiovascular diseases; CKD, chronic kidney disease; SBP, systolic blood pressure; DBP, diastolic blood pressure; eGFR, estimated glomerular filtration rate; ACEI/ARB, angiotensin-converting enzyme inhibitors/angiotensin II receptor blockers; CCB, Calcium Channel Blockers; SCr, Serum creatinine; BUN, blood urea nitrogen; TC, total cholesterol; HDL-C, high density lipoprotein cholesterol; LDL-C, low density lipoprotein cholesterol; FBG, fasting blood glucose.

Patients in Q4 were more likely to be younger males and had a lower proportion of Black individuals (all *p*<0.05). BMI showed a significant upward trend, increasing from 28.36 kg/m² in Q1 to 29.89 kg/m² in Q4 (*p* < 0.001). Similarly, metabolic parameters including TC, LDL-C, TG, and FBG increased progressively across the quartiles, while HDL-C declined significantly (all *p* < 0.001). Specifically, TC increased from 182 mg/dl in Q1 to 202 mg/dl in Q4, LDL-C from 108 mg/dl to 116 mg/dl, TG from 64 mg/dl to 194 mg/dl, and FBG from 92 mg/dl to 103 mg/dl, whereas HDL-C decreased from 59 mg/dl to 43 mg/dl across the same quartiles. Additionally, eGFR decreased progressively from 76.12 ml/min/1.73 m² in Q1 to 73.80 ml/min/1.73 m² in Q4 (*p* < 0.001), with no significant differences in SCr and BUN levels across quartiles.

### Relationship between the TyG index and outcomes

**Figure 2:**
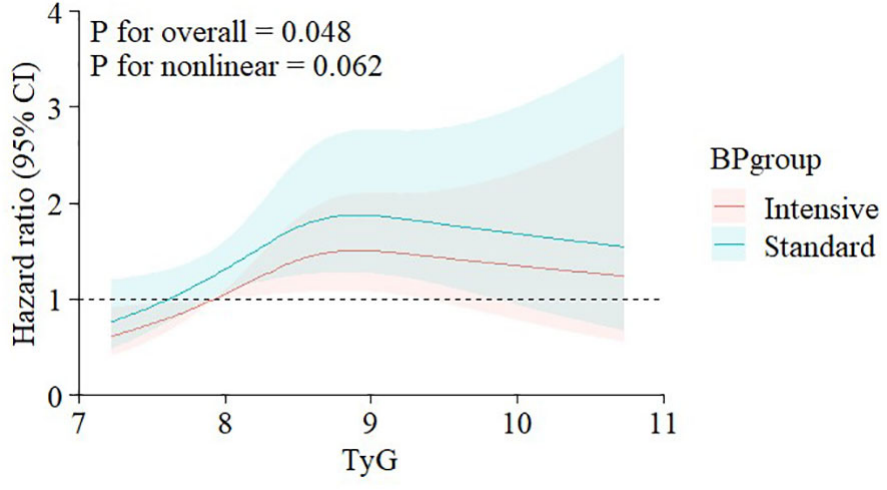
Nonlinear associations of triglyceride-glucose index with CVD events stratified by treatment goal. Graphs show HRs for CVDs adjusted for age, gender, race of black, BMI, CVD history, CKD history, Framingham risk scale, number of antihypertension drugs, use of aspirin, β-blocker, CCB, diuretic, stain, and ACEI/ARB, smoke status, SBP, DBP, eGFR, BUN, HDL-C, LDL-C, FBG. Data were fitted by restricted cubic regression models. Solid lines (red=intensive group, blue=standard group) indicate HRs, and shadow shapes indicate 95% CIs. CVD, cardiovascular disease; HR, hazard ratio; BMI, body mass index; CKD, chronic kidney disease; CCB, calcium channel blocker; ACEI, angiotensin-converting enzyme inhibitor; ARB, angiotensin II receptor blocker; SBP, systolic blood pressure; DBP, diastolic blood pressure; eGFR, estimated glomerular filtration rate; BUN, blood urea nitrogen; HDL-C, high-density lipoprotein cholesterol; LDL-C, low-density lipoprotein cholesterol; FBG, fasting blood glucose; CI, confidence interval.

After a median follow-up of 3.33 years, 725 CVD events were documented. Cox RCS analysis revealed a parabolic dose-response correlation between the TyG index and CVD outcomes, indicating that the association between the TyG index and CVD event risk was not significantly nonlinear, even after adjusting for covariates (*p* for nonlinearity > 0.05, **Figure 2**). Both univariate and multivariate Cox regression analyses, detailed in **Supplementary Table S1** of the **Supplementary Appendix**, identified the TyG index as an independent predictor of CVD outcomes.

**Figure 3:**
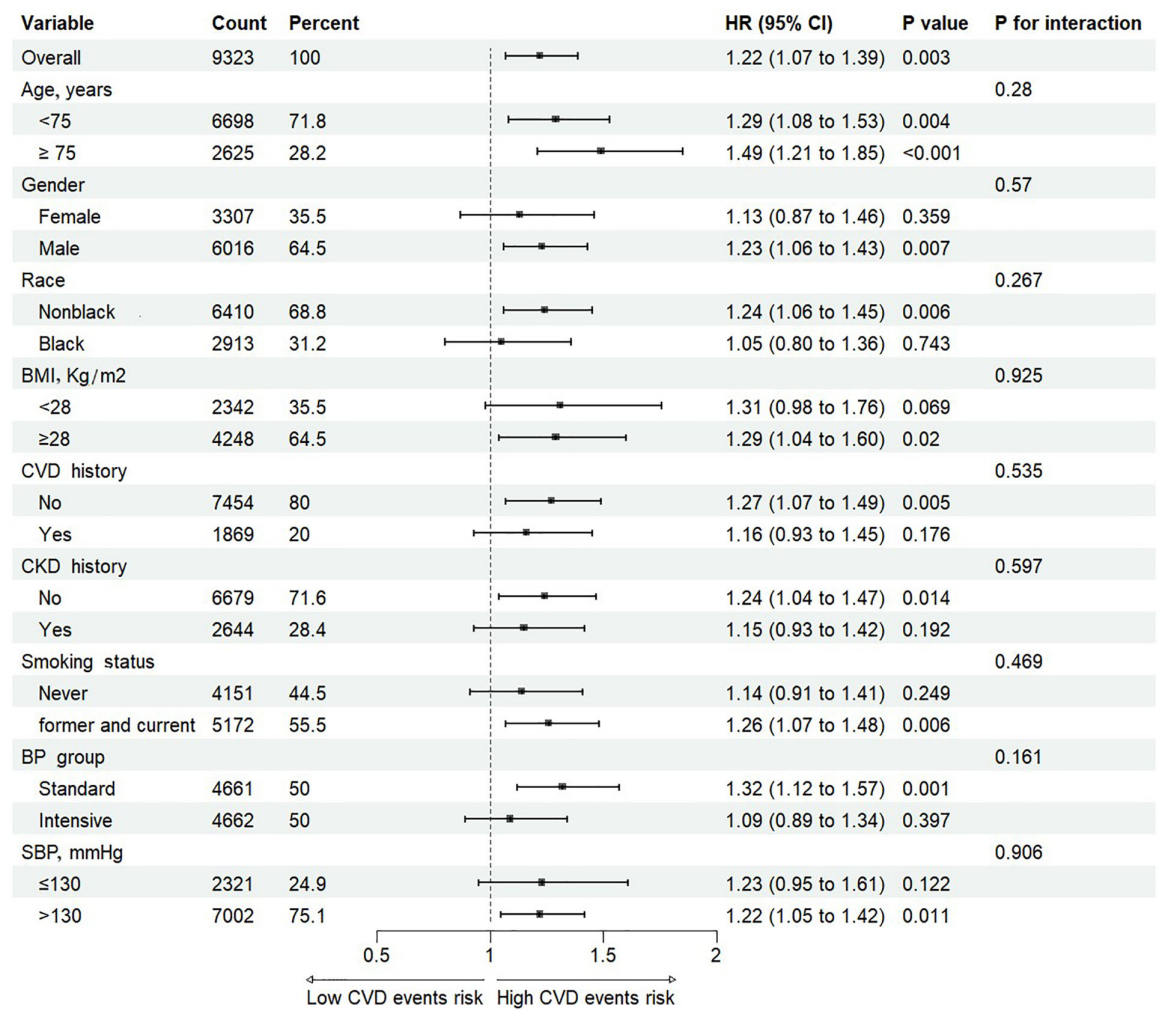
Cox proportional hazards analysis evaluating prognostic implication of TyG index in various subgroups. HR was evaluated by 1-SD increase of TyG index. TyG, triglyceride-glucose; HR, hazard ratio; SD, standard deviation; BMI, body mass index; CKD, Chronic kidney disease; CVD, cardiovascular disease; SBP, systolic blood pressure.

Subgroup analyses evaluated the associations between the TyG index and CVD events, stratified by age (<75 or ≥75 years), gender, race (Black or non-Black), BMI (<28 or ≥28 kg/m²), history of CVD, history of CKD, smoking status (current/former or never), BP treatment group (intensive or standard), and SBP status (<130 or ≥130 mmHg). No significant interactions were found between the TyG index and any of these factors (all *p* values for interactions >0.05, **Figure 3**). This indicates that the proportional effect of a higher TyG index on CVD event outcomes was consistent across all pre-specified subgroups.

**Figure 4:**
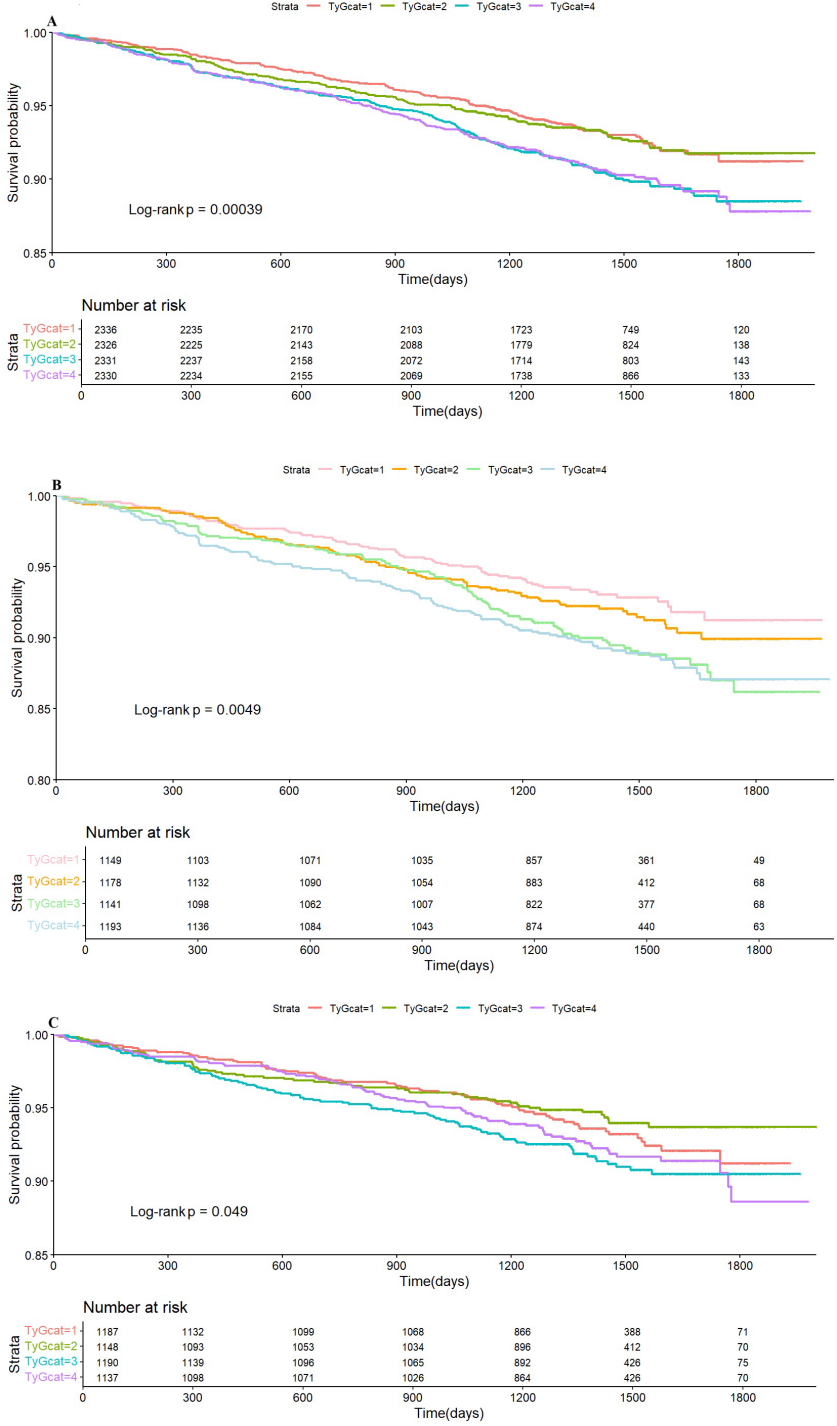
Kaplan–Meier survival analysis curves for the whole cohort **(A)**, standard group **(B)**, and intensive group **(C)** stratified by quartiles of the TyG index. TyG, triglyceride glucose index.

Subsequently, Kaplan–Meier curve analysis was performed to investigate the prognostic impact of the TyG index on CVD endpoints across the entire cohort as well as within distinct BP treatment groups. The analysis revealed that patients in the higher quartiles of the TyG index (Q3 and Q4) were significantly associated with a higher risk of CVD outcomes in the entire cohort (**Figure 4A**) and the standard treatment group (**Figure 4B**) (*p <*0.05) compared to those in the lower quartiles (Q1 and Q2). However, in the intensive treatment group, the difference was statistically significant (**Figure 4C**, p =0.049).

### TyG index as a predictor of CVD risk across BP treatment groups

**Table 2** depicts the associations between the TyG index and the risk of CVD outcomes across various BP treatment groups. Multivariate Cox proportional hazards regression analysis revealed that the TyG index consistently predicted outcomes in the standard treatment group, irrespective of confounder adjustments. However, this trend became less pronounced in the intensive treatment group. Specifically, when the TyG index was treated as a continuous variable, each SD increase in the TyG index within the standard treatment group corresponded to a 32%, 57%, and 40% higher risk of incident primary endpoint events in models 1, 2, and 3, respectively. Analyzing the TyG index as a categorical variable, the unadjusted HR for CVD endpoint events compared to the reference Q1 was 1.50 (95% CI 1.12–2.00) in Q3 and 1.57 (95% CI 1.18–2.08) in Q4 in Model 1. The risk of CVD events significantly increased from Q1 to Q4 (*p* for trend = 0.001). A similar pattern was observed in partially adjusted Model 2 (per SD increase: HR = 1.57, 95% CI 1.31–1.87; Q3: HR = 1.63, 95% CI 1.22–2.19; Q4: HR = 1.92, 95% CI 1.44–2.57; *p* for trend < 0.001) and fully adjusted Model 3 (per SD increase: HR = 1.40, 95% CI 1.09–1.79; Q3: HR = 1.44, 95% CI 1.04–1.98; Q4: HR = 1.64, 95% CI 1.15–2.34; *p* for trend = 0.04). However, The TyG index was not significantly associated with CVD event risk in the intensive treatment group. The increased risk of CVD events per SD or from Q1 to Q4 was solely observed in Model 2 (*p* for trend = 0.002) (**Table 2**), the association between TyG quartiles and CVD risk was attenuated and no longer statistically significant without adjustment (p for trend=0.083) or after full adjustment (p for trend=0.07). No interaction was observed between the two groups across all three models (*p* for interaction > 0.05).

**Table 2:** Association between TyG and CVD endpoint events (Cox regression).

TyG	Intensive group		Standard group		*p* for interaction
	HR (95% CI)	*p* value	HR (95% CI)	*p* value	
Model 1					
Per 1 SD increase	1.09 (0.89-1.34)	0.40	**1.32 (1.12-1.57)**	**0.001**	0.16
6.74≤TyG<8.21	Reference		Reference		
8.21≤ TyG <8.55	0.85 (0.61-1.20)	0.36	1.18 (0.87-1.59)	0.29	
8.55≤ TyG <8.93	1.30 (0.96-1.76)	0.09	**1.50 (1.12-2.00)**	**0.01**	0.48
8.93≤TyG<12.47	1.19 (0.87-1.63)	0.28	**1.57 (1.18-2.08)**	**0.001**	
P for trend	0.083		**0.001**		
Model 2					
Per 1 SD increase	**1.30 (1.05-1.61)**	**0.02**	**1.57 (1.31-1.87)**	**<0.001**	0.21
6.74≤TyG<8.21	Reference		Reference		0.51
8.21≤ TyG <8.55	0.90 (0.64-1.27)	0.55	1.25 (0.92-1.69)	0.15	
8.55≤ TyG <8.93	**1.44 (1.06-1.96)**	**0.02**	**1.63 (1.22-2.19)**	**0.001**	
8.93≤TyG<12.47	**1.50 (1.09-2.07)**	**0.01**	**1.92 (1.44-2.57)**	**<0.001**	
P for trend	**0.002**		**<0.001**		
Model 3					
Per 1 SD increase	1.15 (0.86-1.53)	0.35	1.40 (1.09-1.79)	0.009	0.40
6.74≤TyG<8.21	Reference		Reference		
8.21≤ TyG <8.55	0.86 (0.60-1.263)	0.40	1.20 (0.87-1.639)	0.29	
8.55≤ TyG <8.93	1.30 (0.92-1.83)	0.13	**1.44 (1.04-1.98)**	**0.03**	0.56
8.93≤TyG<12.47	1.26 (0.85-1.86)	0.70	**1.64 (1.15-2.34)**	**0.02**	
P for trend	0.07		**0.04**		

The **bold font** indicates significance at the *p* < 0.05 level.

Model 1 was unadjusted.

Model 2 was adjusted for age, gender, and race.

Model 3 was adjusted age, gender, race of black, BMI, CVD history, CKD history, Framingham risk scale, number of antihypertension drugs, use of aspirin, β-blocker, CCB, diuretic, stain, and ACEI/ARB, smoke status, SBP, DBP, eGFR, BUN, HDL-c, LDL-c, FBG.

TyG index, triglyceride–glucose index; BMI, body mass index; CVD, cardiovascular disease; CKD, chronic kidney disease; CCB, calcium channel blocker; ACEI, angiotensin-converting enzyme inhibitor; ARB, angiotensin II receptor blocker; SBP, systolic blood pressure; DBP, diastolic blood pressure; eGFR, estimated glomerular filtration rate; BUN, blood urea nitrogen; HDL-C, high-density lipoprotein cholesterol; LDL-C, low-density lipoprotein cholesterol; FBG, fasting blood glucose.

## Discussion

This study examined the link between IR, as indicated by the TyG index, and the incidence of CVD events within the SPRINT cohort. Our finding are as follows: (1) The TyG index was independently associated with the occurrence of CVD events in individuals with controlled hypertension. (2) Intensive BP therapy has not yet been proven superior to standard treatment in terms of reducing the risk of CVD events associated with high IR, although a stronger association between the TyG index and CVD events was found in the standard treatment group.

Recent studies have demonstrated that the TyG index correlates with the euglycemic-hyperinsulinemic clamp test and serves as a valid surrogate for the HOMA-IR. Owing to its accessibility and effectiveness, the TyG index is an appropriate and straightforward surrogate for IR, making it suitable for large-scale epidemiological studies and *post-hoc* analyses of randomized controlled trials. The TyG index is a reliable predictor for CVD risk stratification (4, 19) and an effective prognostic indicator in the general population (20, 21). Additionally, the TyG index is strongly associated with the incident of CVD events and prognoses among individuals with conditions such as CVDs (8, 22), hypertension (23, 24), coronary heart disease (25), heart failure (26, 27), and stroke (28, 29). Growing evidence suggests that intensive BP reduction may confer greater cardiovascular benefits; however, the extent to which these benefits are influenced by IR remains unclear. In this study, we utilized the TyG index as a surrogate marker for IR to retrospectively evaluate the associations between varying TyG index values and cardiovascular outcomes in both the intensive and standard treatment groups of the SPRINT trial. Our analysis revealed that patients in the upper quartiles of the TyG index tended to be younger, more frequently obese, and exhibited worse metabolic markers, indicating an elevated risk of MS. Data from the US National Health and Nutrition Examination Survey (2011-2016) suggested a notable rise in MS prevalence among young adults, although the highest prevalence remained among individuals aged 60 years or older (30). Meanwhile, previous research has suggested that the inflammation accompanying excess adiposity may be particularly relevant for coronary heart disease (CHD) occurring at younger ages. Furthermore, most lipid, metabolic, and inflammatory biomarkers showed significant associations with CVD in participants under 55 years, with these associations attenuating with increasing age at onset (31). Moreover, a higher percentage of these patients took β-blockers and diuretics, and had poor renal function, which can affect glucose and lipid metabolic markers. Women generally have a higher CVD risk (32, 33), but in our study, females presented a lower IR, and being female appeared to be a protective factor against CVD events than being male. The protective effect of estrogen and the imbalanced gender ratio in this study should be considered. However, as the SPRINT trial was not designed to evaluate hormonal factors, this hypothesis remains speculative and requires confirmation in future research.

Multiple factors, including C-reactive protein, BMI, and BP level, mediate the relationship between the TyG index and CVD incidence. (12, 34, 35). BP has been shown to partially mediate the correlation between IR and the incidence of cerebral small vessel disease (11) and cardiovascular disease (12). The SBP status significantly influenced the relationship between TyG index and CVD events incidence, with individuals having SBP < 130 mmHg exhibiting a higher risk of CVD events than those with SBP > 130 mmHg (12). Furthermore, the TyG index level is a more significant predictor of all-cause mortality related to CVD events in individuals with controlled hypertension compared to those with uncontrolled hypertension (23). However, persistent high BP status can exacerbate adverse cardiovascular outcomes, potentially confounding the accurate assessment of the TyG index’s prognostic value. Exploring the relationship between the TyG index and CVD events in a controlled hypertension cohort is essential. We first explored the relationship between the TyG index and outcome event risk in patients with controlled hypertension and across different treatment groups. Our study confirmed that the TyG index was still associated with the occurrence of CVD events in patients with controlled hypertension, and this association was independent of other risk factors. Consistent with previous studies, our study also demonstrated a nonlinear correlation between the TyG index and CVD outcomes, though this relationship lacked statistical significance, it warrants further investigation in larger or more diverse cohorts.

We further explored whether intensive BP control could lower the risk of cardiovascular events linked to high IR levels. Contrary to standard BP control, the TyG index was not significantly associated with CVD events in intensive group after comprehensive adjustment for covariates including metabolic markers and renal function. We found a stronger association between the TyG index and CVD events in the standard treatment group, but it must be emphasized that no statistically significant interaction was found in any of the models (p for interaction > 0.05). This highlights the complex interplay and potential confounding factors. The trend may suggest a potential benefit of intensive BP control on TyG-associated risk, further studies are needed to elaborate the specific interactions.

Dysregulation of glucose metabolism and IR may be the critical determinants underlying the variability in CVD benefits derived from intensive BP lowering across different studies. The ACCORD study indicated that patients with DM did not benefit from intensive BP control (SBP < 120 mmHg) in terms of reducing the cardiovascular events risk (13). However, the STEP study, which included 19.1% of DM patients, reached a different conclusion: intensive treatment (SBP 110–130 mmHg) led to a lower incidence of CVD events compared to standard treatment (SBP 130–150 mmHg) among patients aged 60 to 80 years (15). The recently published ESPRIT study included approximately 40% of patients with DM. For high cardiovascular risk hypertensive patients, stricter blood pressure control targets (SBP < 120 mmHg compared with < 140 mmHg) further prevent major vascular events, regardless of their diabetes status (36). The SPRINT study did not included diabetes patients, and further analysis indicated that intensive BP control might elevate the risk of developing new-onset diabetes and impaired fasting glucose e (16). However, the diagnosis of DM relied on self-reports without glycated hemoglobin level confirmation, leaving the severity of DM indeterminate. Moreover, the intensive BP group contained a higher proportion of patients using β-blockers, thiazides, and potassium-sparing diuretics, which can interfere with glucose metabolism. These factors may affect the relationship between intensive BP control and the incidence of new-onset DM. Previous research indicates a positive association between HOMA-IR and SBP in individuals without diabetes and those with prediabetes, independent of antihypertensive medications and abnormal BMI (37). It should be noted that the higher prevalence of β-blocker, stain, and diuretic use in participants with higher TyG index, agents known to potentially influence glucose/lipid metabolism, warrants consideration and reminds us to conduct clinical management of related risk factors.

Elevated BP is linked to a higher risk of DM. In contrast, controlled hypertension can mitigate hyperinsulinemia, reduce the development of insulin-resistant states like DM, and lower CVD risk in MS. Another *post-hoc* analysis of the SPRINT trial found that the cardiovascular benefits of intensive SBP control (SBP <120 mmHg) were consistent regardless of baseline MS status or race/ethnicity (38). In contrast, the JATOS study found that in hypertensive patients under 75 years old, MS was linked to increased CVD risk, and maintaining strict BP control (systolic BP <140 mmHg) was advantageous for those with MS (39, 40). These findings suggest that a lower SBP target (SBP <120 mmHg vs. SBP <140 mmHg) may be more effective in reducing CVD risk of MS patients. Apart from intensive antihypertensive therapy, lifestyle interventions are also central to the treatment of MS. Improvements in the carbohydrate quality index can significant reduced the cardiovascular risk factors, including the TyG index, BP, weight, FBG, and lipids (41). Behavioral interventions (e.g., weight loss, reduced sodium intake, increased physical activity, and limited alcohol consumption) and the DASH diet can significantly improve IR and lower BP. The presence of MS appeared to attenuate these benefits, though the difference was not statistically significant (SBP reduction 9.8 mmHg vs. 11.2 mmHg, *p* = 0.231) (42)

Nonlinear correlations (“U” or “J” shape) between the TyG index and both all-cause and cardiovascular mortality have been documented (9, 10, 21). However, our study identified an almost nonlinear, inverted ‘V’-shaped correlation between the TyG index and CVD events (*p* for nonlinearity = 0.062). SBP status can modify the associations between the TyG index and incident CVD (12). Our study indicates that patients with a higher TyG index may benefit from controlled BP, potentially reducing CVD risk.

Our study, for the first time in a well-designed randomized controlled trial, demonstrates that intensive BP control may attenuate the CVD risk associated with elevated TyG index, though this association was not fully independent of other covariates in the intensive group. Our finding also underscores the importance of assessing both BP levels and IR status of hypertension to identify those at high risk for CVD events. BP management combined with comprehensive lifestyle changes (diet and behavioral interventions) are needed in patients with higher TyG index. Nevertheless, further investigations are needed to confirm these findings.

This study has several limitations that warrant consideration. First, the median 3.33-year follow-up for assessing long-term CVD outcomes has potential limitations. And we lack the TyG index trajectory over time to assess whether there was any improvement in IR under conditions of controlled BP. Second, we did not compare the TyG index with HOMA-IR or the hyperinsulinemic-euglycemic clamp test. Third, lifestyle factors or sociodemographic information, such as education level, marital status, or urban residence, which have been mentioned in other studies, may have influenced the study results. Then, we didn’t perform and report a sensitivity analysis accounting for competing risks to strengthen the robustness of the primary CVD outcome findings, further research is needed in the future to explore this issue. Finally, given the SPRINT trial excluded individuals with diabetes patients and just focused on hypertensive individuals in the U.S., the generalizability of our findings is limited. Future studies are needed to validate these associations in serial TyG measurements and broader populations, including those with diabetes and individuals from diverse racial and ethnic backgrounds.

## Conclusion

For the first time, we revealed the associations between IR, as measured by the TyG index, and CVD events among patients with controlled hypertension. We demonstrated that the TyG index was independently associated with CVD events in the SPRINT cohort, and this association persisted even after adjusting for other established cardiovascular risk factors. Compared with intensive BP control, standard BP treatment showed a stronger association between the TyG index and CVD events. But we do not have definite evidence to prove that intensive BP control is superior to standard antihypertensive treatment in reducing the risk of CVD events associated with higher IR. The TyG index may offer a simple and accessible risk stratification tool in non-diabetic hypertensive patients, and remind us that more attention should be paid to risk factors (e.g., BP status, glucose/lipid metabolism) management of hypertension with a higher TyG index.

## Data Availability

The datasets presented in this study can be found in online repositories. The names of the repository/repositories and accession number(s) can be found below: https://biolincc.nhlbi.nih.gov/studies/sprint/?q=SPRINT.
