# Supplementary material for: Interplay between triglyceride-glucose index and cardiovascular events in hypertensive patients with controlled blood pressure (SBP<140 mmHg): insights from the SPRINT trial
Source: Front Endocrinol (Lausanne). 2025 Jul 16;16:1508667. doi: 10.3389/fendo.2025.1508667 (PMC12307150; doi:10.3389/fendo.2025.1508667)
Supplement: Supplementary file 1 [file Table1.docx]

**Supplementary Appendix**

Table.S1 Univariate and multivariate Cox regression analyses between triglyceride-glucose index and cardiovascular disease outcomes

| **Variables** | **Univariate Cox regression** | | **Multivariate Cox regression** | |
| --- | --- | --- | --- | --- |
|  | **95%CI** | ***p* value** | **95%CI** | ***p* value** |
| Age(years) | 1.052(1.044-1.060) | <0.001 | 1.05(1.03-1.06) | <0.001 |
| Male sex -no. (%) | 1.322(1.128-1.550) | 0.001 | 1.26(1.05-1.53) | 0.016 |
| Race of lack-no. (%) | 0.771(.653-0.910) | 0.002 |  |  |
| CKD history yes | 1.863(1.607-2.159) | <0.001 |  |  |
| CVD history yes | 2.549(2.193-2.963) | <0.001 | 1.92(1.63-2.27) | <0.001 |
| Framingham Risk Scale | 1.150(1.118-1.184) | <0.001 |  |  |
| Intensive treatment- no. (%) | 0.758(0.654-0.878) | <0.001 | 0.74(0.64-0.86) | <0.001 |
| BMI, kg/m^2^ | 0.987(0.974-1.000) | 0.045 |  |  |
| Smoke status |  |  |  |  |
| Current | reference |  |  |  |
| Former | 0.96(0.78-1.2) | 0.74 | 0.77(0.61-0.99) | 0.04 |
| Never | 0.74(0.59-0.92) | 0.01 | 0.68(0.541-0.87) | 0.002 |
| SBP | 1.006(1.001-1.010) | 0.014 |  |  |
| DBP | 0.980(0.974-0.987) | <0.001 |  |  |
| Number of drugs | 1.28(1.19-1.37) | <0.001 |  |  |
| ACEI/ARB | 1.060(0.914-1.229) | 0.442 |  |  |
| β-blocker | 2.030(1.755-2.349) | <0.001 | 1.46(1.13-1.77) | 0.002 |
| α-blocker | 1.280(0.995-1.645) | 0.054 |  |  |
| CCB | 1.267(1.092-1.470) | 0.002 |  |  |
| diuretic | 0.875(0.755-1.015) | 0.079 |  |  |
| Stain | 1.034(0.872-1.227) | 0.699 |  |  |
| Aspirin | 1.320(1.139-1.530) | <0.001 |  |  |
| eGFR (ml/min/1.73 m^2^) | 0.983(0.980-0.987) | <0.001 |  |  |
| SCr, mg/dl | 2.283(1.949-2.675) | <0.001 |  |  |
| BUN, mg/dl | 1.042(1.034-1.051) | <0.001 | 1.02(1.01-1.03) | 0.003 |
| Fasting TC, mg/dl | 0.997(0.995-0.998) | <0.001 |  |  |
| Fasting TG, mg/dl | 1.001(1.000-1.001) | 0.047 |  |  |
| Fasting HDL-C, mg/dl | 0.990(0.985-0.996) | <0.001 |  |  |
| Fasting LDL-C, mg/dl | .0996(0.994-0.998) | <0.001 |  |  |
| FBG, mg/dl | 1.001(0.996-1.007) | 0.617 |  |  |
| TyG | 1.222(1.071-1.393) | 0.003 | 1.28(1.06-1.54) | 0.01 |
| 6.74≤TyG＜8.21 | Reference |  |  |  |
| 8.21≤ TyG ＜8.55 | 1.025(0.819-1.283) | 0.829 | 1.03(0.82-1.31) | 0.79 |
| 8.55≤ TyG ＜8.93 | 1.398(1.134-1.724) | 0.002 | 1.36(1.08-1.73) | 0.009 |
| 8.93≤TyG＜12.47 | 1.390(1.128-1.715) | 0.002 | 1.45(1.12-1.89) | 0.005 |

TyG index, triglyceride–glucose index; CKD, chronic kidney disease; CVD, cardiovascular disease; BMI, body mass index; SBP, systolic blood pressure; DBP, diastolic blood pressure; ACEI, angiotensin-converting enzyme inhibitor; ARB, angiotensin II receptor blocker; CCB, calcium channel blocker; eGFR, estimated glomerular filtration rate; BUN, blood urea nitrogen; TC, total cholesterol; TG, triglyceride; HDL-C, high-density lipoprotein cholesterol; LDL-C, low-density lipoprotein cholesterol; FBG, fasting blood glucose.
